# Retrospective study of factors associated with the clinical severity of covid-19 in older adults in Minas Gerais: structural equation modeling

**DOI:** 10.1590/1516-3180.2023.0138.R1.03072024

**Published:** 2024-12-20

**Authors:** Flavia Aparecida Dias Marmo, Nayara Gomes Nunes Oliveira, Érica Midori Ikegami, Neilzo Nunes Oliveira, Joilson Meneguci, Darlene Mara dos Santos Tavares

**Affiliations:** I Associate Professor, Department of Nursing Education and Community Health, Nursing Graduate Program, Universidade Federal do Triângulo Mineiro (UFTM), Uberaba (MG) Brazil.; II Specialist in older people health, Clinical Hospital, Universidade Federal de Uberlândia (UFU), Uberlândia (MG), Brazil.; III Postgraduate Program in Health Care, Universidade Federal do Triângulo Mineiro (UFTM), Uberaba (MG), Brazil.; IV Clinical Hospital, Universidade Federal de Uberlândia (UFU), Uberlândia (MG), Brazil.; V Postgraduate Program in Physical Education, Clinical Hospital, Universidade Federal do Triângulo Mineiro (UFTM), Uberaba (MG) Brazil.; VI Full Professor, Department of Nursing Education and Community Health, Nursing Graduate Program, Universidade Federal do Triângulo Mineiro (UFTM), Uberaba (MG), Brazil.

**Keywords:** Aged., COVID-19., SARS-CoV-2., Models., Statistical., Older adults., New coronavirus., Pandemic.

## Abstract

**BACKGROUND:**

Studies have shown an association between the clinical severity of coronavirus disease (COVID-19) and sociodemographic and clinical variables in older adults. However, few studies have described the explanatory factors of the relationship between these variables and the clinical severity of COVID-19 using structural equation modeling.

**OBJECTIVE:**

To analyze the factors directly and indirectly associated with the clinical severity of coronavirus disease (COVID-19) among older adults in Minas Gerais, Brazil.

**DESIGN AND SETTING:**

Retrospective epidemiological study.

**METHODS:**

This study included 51,141 elderly adults with COVID-19 living in Minas Gerais, Brazil. Data were collected through the Individual Registration Form – Hospitalized Cases of Severe Acute Respiratory Syndrome from January 28, 2020, to January 27, 2022.

**RESULTS:**

Older age (P < 0.001), male sex (P < 0.001), dyspnea (P < 0.001), change in chest X-ray examination findings (P < 0.001), greater number of risk factors/comorbidities (P < 0.001), and longer hospitalization time (P < 0.001) were directly associated with the clinical severity of COVID-19. Female sex, mediated by the greater number of risk/comorbidity factors (β = -0.02, P < 0.001), and younger age, mediated by longer hospitalization time (β = -0.01; P < 0.001), were indirectly associated with the clinical severity of COVID-19.

**CONCLUSION:**

Demographic and clinical variables were directly associated with increased disease severity. In addition to the direct effect, a greater number of risk/comorbidity factors and longer hospitalization time mediated the association between demographic variables and outcomes.

## INTRODUCTION

Population aging is a global phenomenon.^1^ In Brazil, older adults, characterized by age equal to or greater than 60 years, correspond to 13.8% of the population.^2^ Specifically, in the state of Minas Gerais, where the current study was developed, the elderly population represents 15.4%^2^ and has the highest aging rate in the country.^3^

Due to the physiological changes that occur with the human aging process and affect the immune system, as well as the greater number of complications resulting from chronic diseases, the older adults are more vulnerable to the clinical severity of the coronavirus disease 2019 (COVID-19), which includes the highest proportion of admissions to the intensive care unit (ICU), use of invasive ventilatory support, and death,^4,5^ when compared to younger individuals.^4,6^ Corroborating these findings, in Minas Gerais, it was observed that the occurrence of deaths from COVID-19 increased with advancing age, with 22.3% among older adults aged 60 to 69 years, 26.3% from 70 to 79 years, and 29.9% from 80 years or older.^7^

According to studies carried out among older adults, there is evidence of an association between the clinical severity of COVID-19 and advanced age;^6^ the presence of morbidities;^8^ male sex;^9,10^ frailty;^11,12,13^ malnutrition;^14,15,16^ obesity^17^ and clinical and laboratory test alterations related to inflammatory manifestations and deterioration of immune function.^11,18,19^ In this sense, the investigation of these factors contributes to the detection of risk situations, in addition to the implementation of adequate care plans for the health demands of older adults during the COVID-19 pandemic.

However, it is not clear which of these factors acts directly or through mediation, given the scarcity of studies that describe the explanatory factors of the relationship between sociodemographic and clinical variables and the clinical severity of COVID-19 among older adults,^6^ using structural equation modeling models.

Scientific knowledge on the subject in question is limited and structural equation modeling models have the potential to identify the dependence and interaction of multiple variables, as well as to estimate the direct effects and those mediated by other factors that are part of the causal network of the outcomes of interest.^20^ Therefore, this study aims to analyze the factors directly and indirectly associated with the clinical severity of COVID-19.

## OBJECTIVE

The objective of this study was to analyze the factors directly and indirectly associated with the clinical severity of COVID-19 among older adults in the state of Minas Gerais.

## METHODS

A retrospective epidemiological study was conducted using the information obtained through the Individual Record Form – Cases of Severe Acute Respiratory Syndrome Hospitalized for the state of Minas Garais. The data was obtained from a Microsoft Office Excel^®^ version 10 (Redmond, Washington) datasheet available on the Integrated Health Surveillance Platform of the Ministry of Health (http://plataforma.saude.gov.br/coronavirus/dados-abertos/), specifically within the Influenza Epidemiological Surveillance Information System (SIVEP-Gripe).^21^

Data from a two-year period (January 28, 2020, to January 27, 2022) were used, starting from the date of the first notification of suspected COVID-19 in the state. The study included hospitalized adult patients aged 60 years or older with confirmed COVID-19 (form classification: option 5 - severe acute respiratory syndrome (SARS) due to COVID-19). Cases with incomplete data were excluded as they did not meet the study’s criteria.

**Figure 1:**
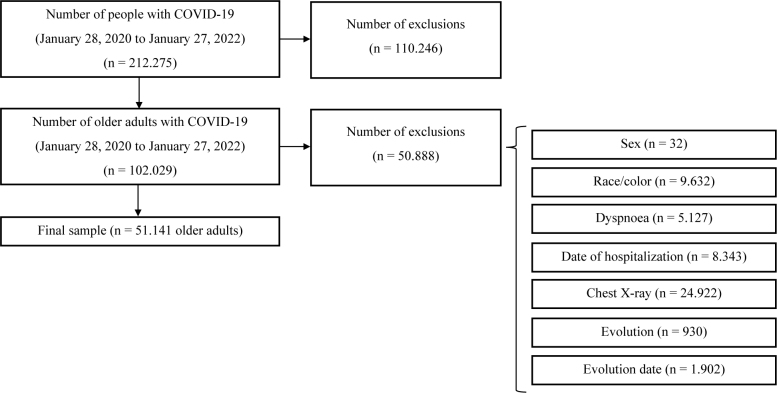
Sample composition.

Of the total number of COVID-19 cases diagnosed in the state of Minas Gerais in the two-year period (n = 212,275), 102,029 were hospitalized older adults. Of these, 50,888 were excluded due to incomplete data (**Figure 1**). Thus, a total of 51,141 older adults met the established criteria and constituted the final sample for the study (**Figure 1**).

The independent variables were demographic: sex (female; male), age (mean years of complete life), color/race (white; non-white); and clinical: risk factors/comorbidity: chronic cardiovascular disease; chronic hematologic; chronic liver disease; asthma; diabetes mellitus; chronic neurological; chronic lung disease; immunodeficiency/immunosuppression; chronic renal; obesity; others (mean number of risk factors/comorbidity); chest X-ray (normal; abnormal); dyspnea (yes; no) and length of hospital stay (mean number of days of hospitalization). The dependent variable, which was defined based on previous studies, was the clinical severity of COVID-19, which included the use of ventilatory support (yes or no), ICU stay (yes or no), and death (yes or no).^4-5^

After selecting the variables, the database was imported to the SPSS (version 24.0; IBM Corp, Armonk, New York, United States) and Analysis of Moment Structures (AMOS), version 24.0 (Armonk, New York, United States).

Data were subjected to descriptive analysis using absolute and relative frequencies for categorical variables, and means and standard deviations for quantitative variables.

**Figure 2:**
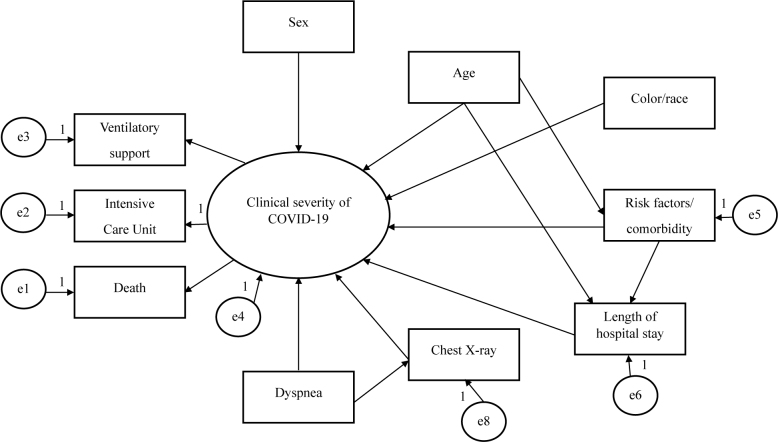
Hypothetical model.

For the construction of the structural model, based on the scientific literature, it was considered that demographic and clinical characteristics were associated with the clinical severity of COVID-19,^4,5^ through direct and indirect trajectories. Thus, a hypothetical model was developed (**Figure 2**) and tested through the analysis of trajectories composed of observed variables, represented by rectangles, and classified as endogenous or exogenous. Endogenous variables receive directional arrows and measurement errors are attributed, specified by the letter “e” in the models.^20^

From the hypothetical model (**Figure 2**), the steps for the analysis of structural equation modeling were as follows: data collection, model estimation, and evaluation of the goodness of fit. The parameters were estimated by the Free Asymptotic Distribution method and the fit qualities of the models were evaluated according to: Chi-square test (χ^2^) P > 0.05; goodness of fit index (GFI) ≥ 0.90; comparative fit index (CFI) ≥ 0.90 and root mean error of approximation (RMSEA) ≤ 0.05.^20^ The model was tested and, subsequently, respecifications were performed (elimination of non-significant pathways (P > 0.05) and calculations of modification indices (≥ 11)).^20^

Direct associations were presented by the estimates of the standardized coefficients of the trajectories between demographic variables, health conditions, and clinical severity of COVID-19. Indirect standardized coefficients were obtained by multiplying the coefficients of the direct paths between variables, and significance was evaluated using the Goodman test. In all tests, the type I error was fixed at 5% (P value < 0.05).^20^

Submission to and approval by an ethics committee was not necessary, given that the present study analyzed the data without identifying registered cases. These are open-access and freely available public secondary data.^21^

## RESULTS

Among the older adults included in the study, there was a predominance of men (50.8%), self-reported non-white skin color/race (51.3%), normal chest radiographs (60.6%), and dyspnea (77.8%) (**Table 1**). Regarding variables related to the clinical severity of COVID-19, the majority used ventilatory support (82.5%), were not admitted to the ICU (63.9%), and were discharged from the hospital (54.4%). However, it is important to emphasize that 45.6% of older adults died (**Table 1**).

**Table 1:** Distribution of demographic variables, health conditions, and clinical severity, included in the model among older adults diagnosed with COVID-19 in a two-year period (2020-2022) in the state of Minas Gerais, Brazil, 2022

Variables	n = 51,141	%	Mean	Standard deviation
Sex				
Male	25998	50.8	-	-
Female	25143	49.2	-	-
**Age**	-	-	73.46	9.21
**Color/race**				
Non-white	27170	51.3	-	-
White	23971	46.9	-	-
**Risk factors/comorbidity**	-	-	1.49	1.09
**Chest X-ray**				
Normal	31006	60.6	-	-
Abnormal	20135	39.4	-	-
**Dyspnea**				
Yes	39765	77.8	-	-
No	11376	22.2	-	-
**Length of hospital stay**	-	-	12.38	9.48
***Clinical severity of COVID-19***				
**Ventilatory support**				
Yes	42196	82.5	-	-
No	8945	17.5	-	-
**ICU stay**				
Yes	18440	36.1	-	-
No	32701	63.9	-	-
**Death**				
Yes	23313	45.6	-	-
No	27828	54.4	-	-

ICU = intensive care unit; COVID-19 = coronavirus disease 2019.

The distributions of the demographic variables, health conditions, and clinical severity included in the model are shown in **Table 1**.

Older age (P < 0.001), male sex (P < 0.001), report of dyspnea (P < 0.001), change in chest X-ray examination (P < 0.001), greater number of risk factors/comorbidities (P < 0.001), and length of hospital stay (P < 0.001) were directly associated with the clinical severity of COVID-19 (**Table 2**). Furthermore, it was observed that female sex (β = -0.02), mediated by the greater number of risk factors/comorbidity, and younger age (β = -0.01), mediated by longer hospitalization time, were associated with indirectly to the clinical severity of COVID-19 (**Table 2**).

**Table 2:** Direct and indirect standardized estimators for variables associated with clinical severity among older adults diagnosed with COVID-19 in a two-year period (2020-2022) in the state of Minas Gerais, Brazil, 2022

Direct and indirect associations	Estimator	P*
**Direct associations**		
Age → Clinical severity of COVID-19	0.25	< 0.001
Sex → Clinical severity of COVID-19	0.10	< 0.001
Dyspnea → Clinical severity of COVID-19	0.27	< 0.001
Chest X-ray → Clinical severity of COVID-19	0.11	< 0.001
Number of risk factors/comorbidity → Clinical severity of COVID-19	0.22	< 0.001
Length of hospital stay → Clinical severity of COVID-19	0.14	< 0.001
**Indirect associations**		
Sex female → Risk factors/comorbidity → Clinical severity of COVID-19	-0.02	< 0.001
Minor age → Length of hospital stay → Clinical severity of COVID-19	-0.01	< 0.001

* P < 0.05; COVID-19 = coronavirus disease 2019.

The direct and indirect estimators of the associations between the tested variables and clinical severity among older adults diagnosed with COVID-19 in the two-year period (2020-2022) in the state of Minas Gerais are shown in **Table 2**.

**Figure 3:**
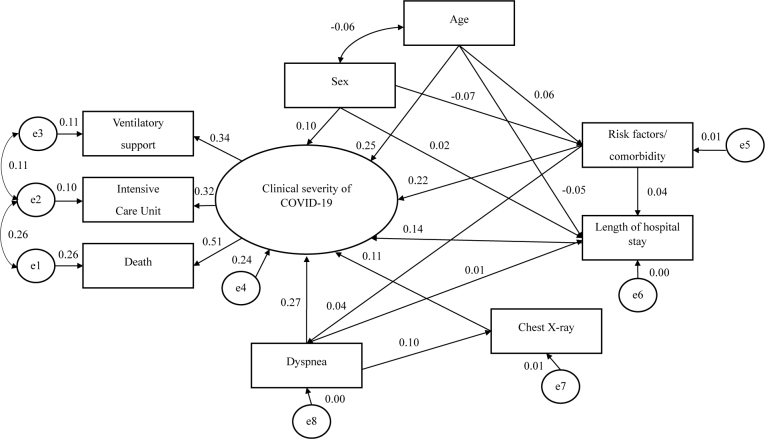
Model with direct and indirect associations between the tested variables and clinical severity among older adults diagnosed with COVID-19 over a two-year period (2020-2022) in the state of Minas Gerais.

Six direct and indirect associations were identified with the clinical severity of COVID-19. The number of risk factors/comorbidities and length of hospital stay were found to have direct and indirect associations with outcomes. Female sex was directly associated with the outcome but was also indirectly associated with risk factors/comorbidities (**Figure 3**).

Fit quality indices (χ^2^ (gl = 16) = 2143,3; P = 0.001; CFI = 0.90; GFI = 0.99; RMSEA = 0.05).

**Figure 3** presents the model with direct and indirect associations between the tested variables and clinical severity among older adults diagnosed with COVID-19 in the two-year period (2020-2022) in Minas Gerais.

## DISCUSSION

The present study shows the factors that directly or indirectly influenced the clinical severity of COVID-19 in the older adults, through structural equation modeling, based on data from the state of Minas Gerais, the second most populous in Brazil.^3^ The factors that were directly associated with the clinical severity of COVID-19 were older age, male sex, greater number of risk/comorbidity factors, longer hospitalization time, altered chest X-rays and the presence of dyspnea. The factors that were indirectly associated were female sex, mediated by a greater number of risk/comorbidity factors, and younger age, mediated by longer hospitalization times.

These results help to understand the factors associated with clinical severity, not only reinforcing that older people, especially men, and those with certain clinical conditions should be prioritized in the implementation and maintenance of preventive measures, but also advance the explanation of factors that can mediate the clinical severity of COVID-19 in women and younger older adults, which can assist in decision-making and public health policy-making.

The association between older age and the clinical severity of COVID-19 corroborates previous research,^23-25^ including advanced age being described as one of the main factors associated with infection by the new coronavirus.^6,24,25^ In the multicenter study HOPE COVID-19, older adults aged 75 years or older had a higher occurrence of death, in addition to the need for oxygen therapy and ICU admission, when compared to the age group of 65 to 74 years.^23^ A systematic review with meta-analysis showed that the proportion of people aged over 65 years was higher in the group of patients with critical and fatal evolution of COVID-19 (Odds Ratio [OR] = 6.01; 95%CI: 3.95-9.16; P < 0.001).^25^

Furthermore, physiological changes inherent to the aging process help to understand, in part, the association between advanced age and clinical severity,^24,26^ as for example, impairment of the immune system and mitochondrial and hormonal functions.^26^ Besides, the presence of underlying comorbidities, prevalent in older adults, can also negatively affect the clinical evolution of the disease.^24,25,26^ These findings confirm the greater vulnerability to the disease associated with age, indicating the need to reinforce preventive measures,^23^ with the aim of minimizing the negative repercussions of COVID-19 in this age group.

Regarding male sex and its association with the clinical severity of COVID-19, a study found that men are more affected by the infection^24^ and are more likely to develop critical conditions, represented by the demand for ventilatory support and ICU admission,^24,25^ in addition to presenting higher mortality when compared to women.^24,25,27^

Similar to advanced age, the association between male sex and the outcome of interest can be explained by immunological characteristics, such as lower responsiveness to infection by the new coronavirus.^24,28^ It is noteworthy that certain diseases and conditions related to severe cases of COVID-19 are more prevalent among men,^25^ such as cardiovascular diseases.^25,26^ In addition to these issues, behavioral factors related to lifestyle among men, such as alcoholism and smoking,^25,28^ and lower adherence to preventive measures, can increase the risk of infections and predispose them to negative outcomes.^28^

International studies have found associations between dyspnea^29^ and alterations in chest X-rays^30^ with the clinical severity of COVID-19 among older adults, corroborating the findings of the present study in which such variables were directly associated with the outcome. Research has shown that patients with a greater extent of alveolar opacities identified on chest radiographs, in general, had a higher mean age,^30^ and more intense respiratory distress,^31^ which is a strong predictor of death.^32^ Such unfavorable circumstances are possibly related to changes in the senescent immune system,^24,26^ and to a higher prevalence or occurrence of morbidities among older adults.^24-26^ Therefore, detailing the characteristics of older adults with a higher risk of adverse outcomes can provide early interventions that postpone the worsening of positive cases.

Multiple comorbidities as a risk for the clinical severity of COVID-19 were also evidenced in a survey carried out in Belo Horizonte-MG, predisposing older adults to a greater chance of death (OR = 1.16); this risk factor has been linked to a worse prognosis for hospitalized patients.^33^ International studies have observed that a greater number of health problems increased the risk of in-hospital mortality among adults and older people (1.8 times the risk in people with ≥ 3 versus no underlying condition),^30^ and also only among older people (1.93 times the risk in people with ≥ 5).^34^ From this fact emerges the need for special attention to individuals with multimorbidity, highlighting chronic diseases, a frequent condition among older adults.

The association between the clinical severity of COVID-19 and the length of stay is consistent with a national study that observed a decrease in survival: on average, 2.3% per day for hospitalization and 3.3% per day for ICU stay. For older adults, the probability of survival reached 50% after the thirteenth day, and, on the twenty-third day, patients aged ≥ 85 years had a survival rate of 24%.^35^ It is noteworthy that hospitalization, especially for people older or with comorbidities affected by COVID-19, is one of the signs of the severity of the disease, as approximately 7.2% of hospitalized patients die.^35^

Despite the scientific literature on the subject^24-25,27^ and current findings showing an association between male sex and the clinical severity of COVID-19, the present study advances our understanding of the repercussions of the disease by showing that the outcome is also associated with female sex, mediated by the presence of risk factors and comorbidities.

The greater life expectancy among women^1^ can expose this population to adverse conditions, such as NCDs,^36^ in agreement with studies that found a higher prevalence of multimorbidity for females in general.^37,38^ In addition, data from the Brazilian Longitudinal Study of Aging (ELSI-Brazil) showed that the prevalence of one or more chronic conditions considered at risk for severe COVID-19 was higher for women (86.4%) than for men (74.3%).^39^

Thus, considering that multimorbidities contribute negatively to the clinical evolution of COVID-19,^24-26^ resulting in financial expenses, it is necessary to invest in the approach, diagnosis, and management of these conditions to better use resources and provide care,^37^ mainly for the female population exposed to multiple diseases, violence, and socioeconomic inequalities.^36^

An association was identified between the clinical severity of COVID-19 in younger older adults based on the mediation of longer hospitalization times. Although the period of hospitalization is often longer in older age groups,^29,40^ a multicenter study found a longer average length of stay (P = 0.05) among older people aged 65-74 years (9.4 ± 7.3 days ) when compared to those aged 75 years or older (8.6 ± 6.9 days).^23^ This fact, found in other investigations,^6,19,33^ is related to immunological issues^19,26^ and the presence of risk factors, such as multimorbidities,^19,23,33^ that require intensive^24^ and prolonged care. However, the results of the present study reinforce the repercussions of longer hospital stay on worse prognoses, including mortality,^32^ regardless of age. Thus, it is essential that health services direct efforts and resources to improve their approach and treatment, with the aim of reducing the negative consequences of longer hospital stays.

The present study has limitations, such as the non-inclusion of aspects related to vaccination; that is, the study scope possibly included older adults who received some dose of the vaccine, which may have influenced the outcome. Another limitation refers to wave peaks, as in these periods’ hospitalizations have been associated with higher mortality, as demonstrated by a national study.^41^ Furthermore, the exclusion of 50,888 cases with incomplete data may have influenced the results, highlighting the importance of completing notification forms.

The strengths of this study include the use of a state database and the performance of structural equation modeling analysis, which allowed us to understand the characteristics and health factors that determine the clinical severity of COVID-19.

## CONCLUSION

Demographic characteristics, such as older age and male sex, and clinical characteristics, including the highest number of morbidities, length of hospital stay, change in X-ray examination, and dyspnea, were directly associated with the clinical severity of COVID-19, that is, ICU admission, use of ventilatory support, or death. Female sex, mediated by a greater number of risk/comorbidity factors, and younger age, mediated by a longer hospitalization time, were indirectly associated with the clinical severity of COVID-19. The results of this study can help identify groups at risk of adverse outcomes and make decisions to prioritize older adults who may need more care owing to COVID-19, considering previous health information.
